# Self-sampling in cervical cancer screening: comparison of a brush-based and a lavage-based cervicovaginal self-sampling device

**DOI:** 10.1186/s12885-016-2246-9

**Published:** 2016-03-15

**Authors:** Liisa Karjalainen, Ahti Anttila, Pekka Nieminen, Tapio Luostarinen, Anni Virtanen

**Affiliations:** Mass Screening Registry, Finnish Cancer Registry, Unioninkatu 22, FI-00130, Helsinki, Finland; Department of Obstetrics and Gynaecology, Helsinki University Central Hospital, Jorvi Hospital, Turuntie 150, Espoo, Finland

**Keywords:** Cervical cancer screening, HPV, Self-sampling, Socio-demographic factors, Acceptability

## Abstract

**Background:**

High coverage and attendance is essential for cervical cancer screening success. We investigated whether the previous positive experiences on increasing screening attendance by self-sampling in Finland are sampler device dependent.

**Methods:**

All women identified to cervical cancer screening in 2013 in 28 Finnish municipalities were randomised to receive a lavage- (*n* = 6030) or a brush type of self-sampling device (*n* = 6045) in case of non-attendance after two invitation letters. Seven hundred seventy non-attending women in the lavage device group and 734 in the brush group received the self-sampling offer. Women’s experiences were enquired with an enclosed questionnaire.

**Results:**

Total attendance in the lavage group increased from 71.0 to 77.7 % by reminder letters and further to 80.5 % by self-sampling. Respective increase in the brush group was from 72.2 to 78.6 % and then to 81.5 %. The participation by self-sampling was 21.7 % (95 % CI 18.8–24.6) in the lavage group and 23.8 % (95 % CI 20.8–26.9) in the brush group. Women’s self-sampling experiences were mainly positive and the sampler devices were equally well accepted by the women.

**Conclusion:**

Our study shows that the lavage device and brush device perform similarly in terms of uptake by non-attending women and user comfort. If self-sampling is integrated to the routine screening program in Finland, either of the devices can be chosen without the fear of losing participants due to a less acceptable device.

## Background

A maximal attendance and coverage of screening is crucial to further reduce the incidence and mortality of cervical cancer. In Finland, the attendance rate in the screening program is currently approximately 70 % [1] and a substantial portion of cervical cancers diagnosed among women in screening ages (30–60 years of age) in Finland are detected among women not attending screening [2–4]. The use of pre-assigned appointment times in invitations and reminder letters increase screening attendance by 6.6–9.4 % [5–9]. Offering high risk human papillomavirus (hrHPV) testing on self-taken samples (self-sampling) to the non-attendees of the routine screening helps to overcome practical and emotional barriers to screening and has the potential to increase screening attendance [5, 9–15]. However, only one study thus far has compared the participation and acceptability of different self-sampling devices in an actual screening setting [16]. Based on the previous result the brush device is non-inferior to the lavage device in these aspects [16].

The main aim of this study was to compare the effects of a lavage- and a brush-type self-sampling device on screening attendance within the routine screening program in Finland. We also compared women’s perceptions and experiences of the self-sampling procedure with these devices.

## Methods

In the Finnish screening program all women aged 30–60 years of age are invited by personal invitations in 5-year intervals. In some municipalities also women aged 25 and/or 65-years of age are included. Primary screening modality in most municipalities is Pap-testing, some use primary HPV-testing.

This study was conducted as a part of routine screening in 28 Finnish municipalities in 2013, including both urban and rural areas. The screening visits were arranged locally but all participating municipalities used the same screening laboratory of the Cancer Society of Finland for the analysis of the samples. All participating municipalities used Pap-testing as a screening modality.

**Fig. 1:**
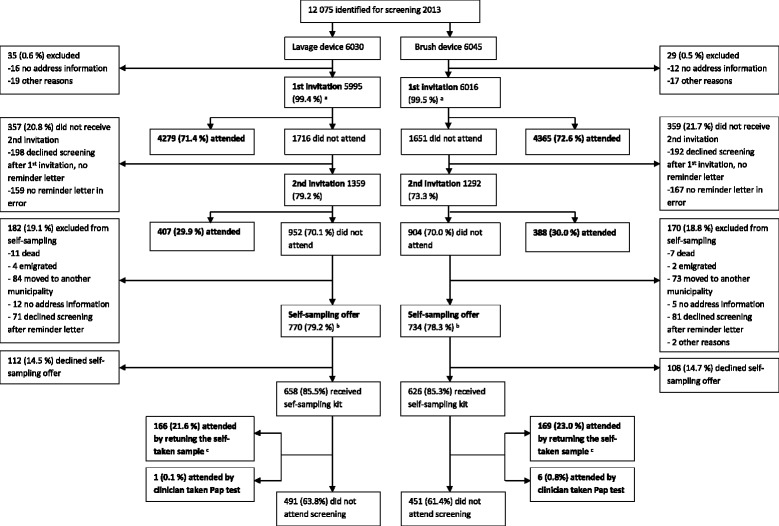
The flow of women in the invitation protocol. ^a^ Includes 324 women in lavage-device arm and 329 women in the brush-device arm who attended before the invitation was sent or made an appointment for screening (e.g., by phone) and thus received no invitation letter. ^b^ Women received an information letter about the up-coming self-sampling test with an opt-out option. ^c^ Out of all women to whom self-sampling was offered to

Originally 32 municipalities (12,555 women) were to take part in the study. Based on previous studies on the use of reminder letters and self-sampling as a second reminder in Finland, we estimated that participation rate after two invitation letters would be 80 and 20 % of women who were offered the lavage-type self-sampling test would participate [5, 6, 17]. This would leave 2511 women (1256 per arm) to be invited by self-sampling and would allow for us to detect a 4.8 % difference in participation rates between self-sampling methods (2 sided, power = 0.8, alpha = 0.05). Later, four municipalities dropped out of the study due to a lack of local resources. Self-sampling groups were also smaller than expected due to missing invitations and e.g., emigration (Fig. 1).

The exact flow of women in the invitation protocol in each research arm is shown in Fig. 1. All women identified for screening in the 28 participating municipalities in 2013 were included in the study. Overall the cohort consisted of 12,075 women who were, upon identification for screening, randomised to receive a lavage-based (6030) or a brush-based self-sampling device (6045) in case of non-attendance. Women were invited to screening by a personal letter. Non-attendees after the primary screening invitation received a second invitation (reminder letter) within the same year - with the exception of those women who had declined from screening altogether when cancelling the given appointment (a feature of the program used when sending out the invitations). Both invitation letters included a pre-assigned appointment time that could be changed by telephone or over the internet. After the two invitation letters, the non-attendees were extracted from the screening database. Only women still living in the original inviting municipality, with address information available, were included in self-sampling intervention, and again women who had declined screening altogether while cancelling the given screening appointment were excluded (Fig. 1). There were a few errors in the invitation protocol. three hundred twenty-six women (159 in the lavage device arm and 167 in the brush device arm) did not receive a reminder letter despite of their non-attendance and two women in the brush device arm were not offered self-sampling test in error (Fig. 1).

Prior to sending out the sampler device, the self-sampling possibility was introduced with a letter with an opt-out option offering women a possibility the decline the self-sampling device beforehand. Questionnaires surveying the women’s experience of sample taking, general attitudes towards self-sampling and previous screening history were sent together with the sampler devices. The women were unaware of the randomisation and the use of two different sampler devices in this study.

The material was sent in the women’s mother tongue (Finnish or Swedish; the two official languages in Finland). Women with mother tongue other than Finnish or Swedish received the material in Finnish with the option to order the material in English. All self-sampling related material was organized centrally by the Mass Screening Registry.

The screening data from the mass screening register was combined to data from Statistics Finland to clarify socio-demographic factors related to the screening participation: mother tongue, education level, marital status and type of home municipality. Education level is recorded in Statistics Finland for those who have completed the lower secondary education. For the purposes of this study we divided the education into three levels: primary (including primary education, currently 9 years in Finland and, due to the registration protocol of Statistics Finland, those with missing education level information), secondary (including lower and upper secondary education) and tertiary (including lower and upper tertiary education and doctoral degree or equivalent). Missing information on education level was updated from the questionnaire answers when possible. The mother tongue was divided into two groups: ‘Finnish or Swedish’, and ‘other’. Women with Finnish or Swedish as mother tongue were combined into one group to avoid too small groups, as they received the study related materials in their own mother tongue and no significant differences in screening behavior have been reported between these groups in previous studies [5, 6, 17]. Marital status was classified into four categories: unmarried, married or widowed, divorced and unknown. Married and widowed women were grouped together to avoid too small groups and because of previously reported similar screening behavior [17]. Statistics Finland divides municipalities into three types – urban, semi-urban and rural – and the same classification was used in this study. With regard to age, the study cohort was divided into 10-year age groups: 30–39 (including also 175 women aged under 30 years), 40–49, 50–59 and 60–69 years of age.

### Self-sampling tests and HPV-analysis

Sample-taking at home was performed by a lavage-based device (Delphi Screener, Delphi Bioscience BV, Sherpenzeel, The Netherlands) or a brush-based device (Evalyn Brush, Rovers medical devices BV, Oss, The Netherlands).

The Delphi Screener produces a lavage-sample by rinsing the cervix and upper vagina with saline. Saline is released by pressing a plunger of the device and flows back into the device when the plunger is released. The Evalyn Brush produces a dry sample by collecting cervical and upper vaginal endothelial cells when the brush is rotated in the upper vagina. The brush device is inserted in the vagina up to its wings, the brush is pushed out from the casing by pressing a plunger and rotated five times [16, 18]. In the laboratory, the cell sample was extracted from the brush by adding buffered saline.

The self-taken samples were analysed using a Hybrid Capture 2 (HC2) assay which detects 13 most common hrHPV types (16, 18, 31, 33, 35, 39, 45, 51, 52, 56, 58, 59, 68) [19]. Only samples producing a visible cell pellet after centrifugation at 1500 rpm were considered adequate. Of the originally returned 166 lavage-type samples one (0.6 %) and of the 170 brush-type samples four (2.4 %) did not fulfill this criteria (the difference not being statistically significant). The women in question received a new device of the same type and the one woman in the lavage device group and three women in the brush device group returned a new adequate sample. In the end 166 lavage-type samples and 169 brush-type samples fulfilled the criteria of an adequate sample and were included in this study. One woman in the lavage device and six women in the brush device group chose to attend screening by Pap smear after receiving the self-sampling kit. They are included in the self-sampling participants by intention-to-treat.

Women were notified of their test result by a letter. Women with a hrHPV-positive result in self-taken sample were either invited for a Pap smear (women <40-years old in 24 municipalities and all women in four municipalities) and referred to a colposcopy in case of a cytological result of low grade squamous cell lesion (LSIL) or more severe, or repeat atypical squamous cells of unknown significance (ASC-US); or referred directly to a colposcopy (women >40-years old in 24 out of 28 municipalities) [20].

### Questionnaire study

In total 1284 questionnaires (658 with the lavage-device and 626 with the brush-device) were sent with the self-sampling kits. The questionnaire was developed based on previous pilot studies and previous literature [15, 21–24]. All questions were presented to an external group of women to ensure clarity. Women gave their written consent to link their answers to the screening data.

Women’s experience on self-sampling was measured using a 16 item survey consisting of 13 questions on sample taking procedure and attitudes towards self-sampling and three on the clarity of the user instructions. A space for open feedback was also provided. Responses to the items were on a five point Likert-type scale from “fully agree” to “fully disagree” and a “cannot say” as options. For the analysis some of the responses were reversed from the original so that “totally agree” would present the most positive experience and maximal acceptability for each of the items. To avoid small response frequencies the answers were grouped into three categories “agree” (fully or somewhat agree), “neither agree nor disagree” and “disagree” (fully or somewhat disagree) for the comparison of the experiences with the sampler devices. Also women’s preferences of the future screening method (self-taken vs. clinician taken test) were enquired.

As opportunistic Pap tests are not registered in any joint database in Finland, the effect on overall screening coverage (including also opportunistic testing) was estimated using data on previous screening history collected with the questionnaire. Women were considered under-screened if they had no previous Pap smears within 5 years.

### Statistical methods

The results were analyzed using Stata 12.1. Age-, mother tongue-, education level-, marital status- and municipality type adjusted relative risks (RRs) and 95 % confidence intervals (CI) for participation by self-sampling were estimated using Poisson regression. Age-, mother tongue-, education level-, marital status- and municipality type adjusted RRs and CIs for the total attendance and for self-sampling participation by brush-device compared to the lavage-device were estimated with logbinomial regression. Student’s paired *t* test was applied to test the increase in total participation by reminder letters and self-sampling in both groups, and Fisher’s exact test to test the statistical significance of the difference in the user experiences between the self-sampling devices.

### Ethics statement

The study was approved by the Ethical committee of the Hospital District of Helsinki and Uusimaa (79/13/03/03/2011) and National Institute for Health and Welfare (THL/1465/6.02.00/2013).

## Results

### Participation by self-sampling

In the lavage group, 167 out of 770 women (21.7 %, 95 % CI 18.8–24.6), and in the brush group, 175 out of 734 women (23.8 %, 95 % CI 20.8–26.9) participated in screening (Table 1).

**Table 1:** The mutually adjusted participation rates after self-sampling offer

	Lavage-device			Brush device			Total			Mutually adjusted total attendance after self-sampling invitation	
	Invited	Attended^a^		Invited	Attended^a^		Invited	Attended^a^		RR	95 % CI
	n	n	%	n	n	%	n	n	%		
Age groups											
30–39	270	48	17,8	277	54	19,5	547	102	18,6	1	
40–49	195	48	24,6	170	47	27,6	365	95	26,0	1,34	1,01–1,78
50–59	193	48	24,9	204	62	30,4	397	110	27,7	1,45	1,10–1,91
60–69	112	23	20,5	83	12	14,5	195	35	17,9	1,03	0,69–1,52
Mother tongue											
Finnish/Swedish	742	161	21,7	700	169	24,1	1442	330	22,9	1	
Other	28	6	21,4	34	6	17,6	62	12	19,4	1,1	0,61–1,95
Education											
Primary	101	8	7,9	105	12	11,4	206	20	9,7	1	
Secondary	341	74	21,7	352	82	23,3	693	156	22,5	2,26	1,41–3,61
Tertiary	328	85	25,9	277	81	29,2	605	166	27,4	2,74	1,71–4,38
Marital status											
Unmarried	259	52	20,1	247	54	21,9	506	106	20,9	1	
Married/Widowed	411	96	23,4	365	95	26,0	776	191	24,6	1,05	0,82–1,34
Divorced	96	19	19,8	114	26	22,8	210	45	21,4	0,98	0,69–1,40
Unknown	4	0	0,0	8	0	0,0	12	0	0,0	0	
Municipality type											
Urban	405	84	20,7	386	88	22,8	791	172	21,7	1	
Semi-urban	196	44	22,4	177	46	26,0	373	90	24,1	1,12	0,87–1,45
Rural	169	39	23,1	171	41	24,0	340	80	23,5	1,14	0,88–1,49
Total	770	167	21,7	734	175	23,8	1504	342	22,7		

^a^Including one woman in the lavage group and six women in the brush group that attended by Pap smear after self-sampling offer

By age, the overall self-sampling participation rate (i.e., with both sampler devices together) was highest among women aged 40–49 and 50–59 years (Table 1). With regard to education level, the participation rate was lowest among women with only primary education and increased significantly with increasing education level. By mother tongue, the crude overall participation rate was slightly higher among Finnish or Swedish speaking women than among women with a mother tongue other than these two, but the difference was not statistically significant in the adjusted model. Further, the difference was seen only in the brush group. The crude participation rates were also higher among married and widowed women, and in semi-urban and rural municipalities, but these differences were not significant in the adjusted model.

Table 2 shows the adjusted relative risk of participation with the brush device in comparison to the lavage device. The participation rate was slightly higher with the brush device in all socio-demographic groups, apart from the oldest age group and women with mother tongue other than Finnish or Swedish, but the differences were non-significant.

**Table 2:** Adjusted relative risks for participation with self-sampling in the brush device group in comparison to the lavage device group

	RR	95 % CI
Total^a^	1.1	0,91–1,32
Age group^b^		
< =39	1.1	0,77–1,58
40–49	1.1	0,76–1,54
50–59	1.28	0,93–1,76
60–69	0.7	0,38–1,29
Mother tongue^c^		
Finnish/Swedish	1.1	0,91–1,33
Other	0.95	0,28–3,20
Education level^d^		
Primary	1.59	0,65–3,91
Secondary	1.05	0,80–1,39
Tertiary	1.11	0,85–1,44
Marital status^e^		
Unmarried	1.17	0,84–1,64
Married/Widowed	1.08	0,85–1,38
Divorced	1.21	0,72–2,06
Municipality type^f^		
Urban	1.15	0,89–1,50
Semi-urban	1.06	0,74–1,53
Rural	1.04	0,71–1,53

In the lavage group four women and in the brush-group eight women with unknown marital status are excluded from the analysis. The women participating with a Pap smear after the self-sampling offer are not included as self-sampling participants in this analysis

^a^Adjusted for age, mother tongue, education level, marital status and municipality type

^b^Adjusted for mother tongue, education level, marital status and municipality type

^c^Adjusted for age, education level, marital status and municipality type

^d^Adjusted for age, mother tongue, marital status and municipality type

^e^Adjusted for age, mother tongue, education level and municipality type

^f^Adjusted for age, mother tongue, education level and marital status

### Increase in total screening attendance

**Fig. 2:**
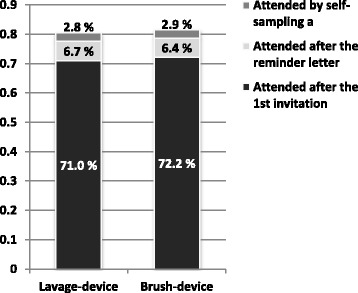
The crude effects of reminder letter and self-sampling on the attendance in the lavage device arm and in the brush device arm. ^a^ Includes one woman in lavage device arm and six women in the brush device arm that attended screening by Pap smear after the self-sampling offer

The participation rate after the primary invitation among all women identified for screening was 71.0 % (95 % CI 69.8–72.1) in the lavage group and 72.2 % (95 % CI 71.1–73.3) in the brush group. The reminder letters increased the attendance to 77.7 % (95 % CI 76.7–78.8) and to 78.6 % (95 % CI 77.6–79.7), respectively. After self-sampling the total attendance reached levels of 80.5 % (95 % CI 79.5–81.5) in the lavage group and 81.5 % (95 % CI 80.5–82.5) in the brush group (Fig. 2). No significant differences in the total attendance rates in different socio-demographic groups were observed between the lavage and brush group (data not shown).

### Effects on screening coverage

Of those non-attendees who participated in screening with a self-taken sample, 64 % (65 % in the lavage group and 64 % in the brush group) reported a Pap smear in the preceding screening interval, i.e., <5 years ago (including also opportunistic screening). Approximately 20 % (24 % in the lavage group and 17 % in the brush group) reported a previous Pap smear over 5 years ago or never and could thus be considered truly under-screened. Only these under-screened self-sampling participants demonstratively increased the overall screening coverage. Approximately 15 % (11 % in the lavage group and 19 % in the brush group) did not answer the question in the questionnaire and their screening history is thus not known.

### Women’s experience of self-sampling

**Fig. 3:**
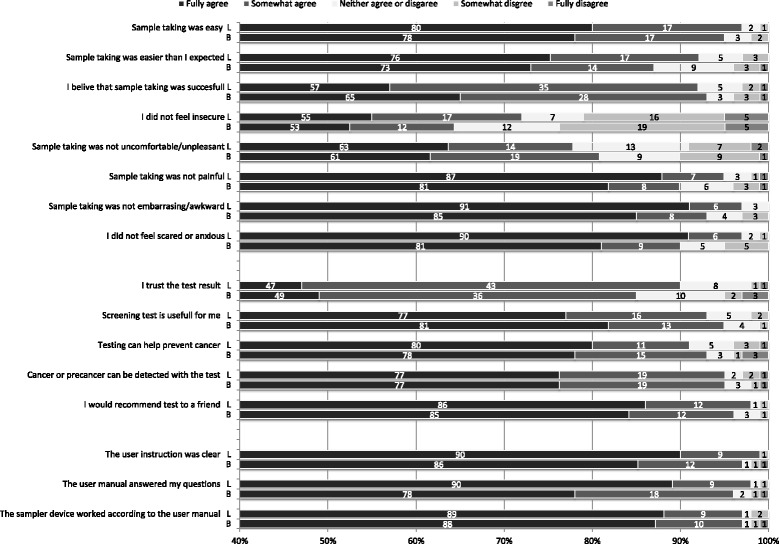
Women’s experience on self-sampling with the lavage device and the brush device. Response frequencies based on the number of completed responses to the sub-question, excluding those who answered “cannot say”. *L* = lavage device, *B* = brush device

Response rates to the questionnaire among the self-sampling participants were 99 % (164/166) in the lavage group and 98 % (165/169) in the brush group. Figure 3 shows women’s responses to the statements addressing their experience on self-sampling. Self-sampling was regarded as easy by 97 % (154/159) of the lavage device users and by 96 % (149/156) of the brush device users who responded to the question (fully or somewhat agree to the statement). Discomfort was reported by 9 % (13/145) by the lavage device users and 10 % (16/153) by the brush device users and pain by 3 % (4/146) and 4 % (6/155), respectively. Feelings of insecurity during sample taking were reported by 21 % (31/145) in the lavage group and 23 % (36/154) in the brush group. In the open answers the most commonly reported problem with the brush device was the concern about the device being inserted in the correct depth for sample taking, and not hearing the clicks when rotating the brush and thus not knowing the number of rotations, but a clear majority did not specify what caused them to feel insecure. The same problems were also reported by the brush users who did not report insecurity. Considering all lavage users who rated their device, the reported problems were related to the plunger of the device not releasing properly, fluid leaking out during sample taking, the volume of the collected sample seeming small and difficulties with the closing strip of the return envelope. The same problems came up among the lavage users who reported insecurity, although most women did not specify the reasons for their insecurity. In both groups several women requested for instructions on how long the sample survives unaffected in a mailbox in the arctic winter conditions.

In both groups 93 % (136/147 in the lavage group and 140/150 in the brush group) of the responders felt confident that the sample was taken successfully. In the lavage group 90 % (135/150) of the responders and in the brush group 85 % (126/149) fully or somewhat agreed that they trust the test results from the self-sampling test. These minor differences in women’s experiences between the samplers among all self-sampling attendees were not statistically significant.

Of the under-screened self-sampling participants (reported last smear >5 years ago or never) 67 women rated their self-sampling experience, 39 in the lavage group and 28 in the brush group. Among these women sample-taking was considered easy (100 % vs. 83 %, *p* = 0.019) and easier than expected (100 vs. 75 %, *p* = 0.003) more often with the lavage device and feelings of insecurity (6 vs. 9 %, *p* = 0.019) and pain (0 % vs 8 %, *p* = 0.03) during sample-taking were reported more often with the brush device (Table 3).

**Table 3:** Self-sampling experiences of the under-screened self-sampling participants with the lavage and the brush device

	Lavage device		Brush device	
	n	%	n	%
Sample taking was easy				
Agree	38	100.0	20	83.3
Neither agree nor disagree	0	0.0	2	8.3
Disagree	0	0.0	2	8.3
*p*-Value*	0.019			
Sample taking was easier than I expected				
Agree	36	100.0	18	75.0
Neither agree nor disagree	0	0.0	4	16.7
Disagree	0	0.0	2	8.3
*p*-Value	0.003			
I believe that sample taking was succesful				
Agree	31	91.2	18	78.3
Neither agree nor disagree	1	2.9	3	13.0
Disagree	2	5.9	2	8.7
*p*-Value	0.385			
I felt insecure				
Agree	31	91.2	18	78.3
Neither agree nor disagree	1	2.9	3	13.0
Disagree	2	5.9	2	8.7
*p*-Value	0.019			
Sample taking was uncomfortable/unpleasant				
Agree	31	88.6	18	72.0
Neither agree nor disagree	3	8.6	3	12.0
Disagree	1	2.9	4	16.0
*p*-Value	0.185			
Sample taking was painful				
Agree	33	100.0	21	84.0
Neither agree nor disagree	0	0.0	2	8.0
Disagree	0	0.0	2	8.0
*p*-Value	0.03			
Sample taking was embarrasing/awkward				
Agree	33	97.1	24	96.0
Neither agree nor disagree	1	2.9	0	0.0
Disagree	0	0.0	1	4.0
*p*-Value	0.672			
I felt scared or anxious				
Agree	34	100.0	22	88.0
Neither agree nor disagree	0	0.0	1	4.0
Disagree	0	0.0	2	8.0
*p*-Value	0.071			
I trust the test result				
Agree	35	92.1	19	79.2
Neither agree nor disagree	3	7.9	3	12.5
Disagree	0	0.0	2	8.3
*p*-Value	0.158			
Screening test is useful for me				
Agree	36	94.7	24	92.3
Neither agree nor disagree	2	5.3	1	3.8
Disagree	0	0.0	1	3.8
*p*-Value	0.741			
Testing can help prevent cancer				
Agree	34	89.5	22	88.0
Neither agree nor disagree	3	7.9	2	8.0
Disagree	1	2.6	1	4.0
*p*-Value	1			
Cancer or precancer can be detected with the test				
Agree	35	92.1	25	96.2
Neither agree nor disagree	3	7.9	0	0.0
Disagree	0	0.0	1	3.8
*p*-Value	0.149			
I would recommend test to a friend				
Agree	37	100.0	24	92.3
Neither agree nor disagree	0	0.0	1	3.8
Disagree	0	0.0	1	3.8
*p*-Value	0.166			

Response frequencies based on the number of completed responses to the sub-question, excluding those who answered “cannot say”

*P values of Fisher’s exact test

### Preference of future screening method

Women were asked which screening method they would prefer in future, self-sampling or traditional clinic based screening. fifty-eight percent (of those who gave answer to the question; 94/163) of women who participated with the lavage device and 66 % (105/159) of women who participated with the brush device would prefer self-sampling in the future. 13 and 7 %, respectively would prefer traditional screening in the future. 28 and 27 %, respectively, had no preference.

### Self-sampling test results

7.2 % (12/166) of the returned lavage-samples and 5.9 % (10/169) of the brush-samples were hrHPV-positive by HC2. 11 women were invited for a Pap smear and 11 referred directly to colposcopy. Of the 11 women invited for a Pap smear, only four (36 %) women attended. All four women had a normal cytology. Of the 11 women referred directly to colposcopy, one did not follow the invitation. The total loss of follow-up was 36 % (8/22). Among women referred to colposcopy, one was diagnosed with CIN3 (cervical intraepithelial neoplasia, dysplasia gravis), five women had a benign finding such as an inflammation or HPV-atypia and four women had normal findings.

## Discussion

The effects of self-sampling as a second reminder, i.e., after a primary invitation and a reminder letter, on total screening attendance did not differ between the lavage and the brush device. Participation with the brush device was slightly higher (23.8 % vs 21.7 %; adjusted RR 1.1, 95 % CI 0.91–1.32), but no significant differences were observed in total attendance after interventions (81.5; 95 % CI 79.5–81.5 vs 80.5 %; 95 % CI 80.5–82.5), or in different socio-demographic groups. Further these self-sampling devices were equally well accepted by the women.

To our knowledge this study exploring the attendance rates and acceptability of two different self-sampling devices among non-attendees to routine screening was the second of its kind. In the previous study from the Netherlands, the self-sampling participation was slightly but significantly higher with the brush device in comparison to the lavage device, the absolute difference being 2.7 %. No hypothesis was presented on the reasons for the higher attendance with the brush device. In our study the non-adjusted absolute difference between the devices was similar, 2.1 %, but did not reach statistical significance due to a smaller study cohort. The Dutch further found no differences in the acceptability of the two devices [16].

The achieved total participation rates in both groups (80.5 % in the lavage group and 81.5 % in the brush group) reached similar levels than in previous Finnish studies with self-sampling used as a second reminder [17]. The previously reported socio-demographic factors related to lower participation rate, observed also in this study, were young age (total participation rate 71 % in this study), a mother tongue other than Finnish or Swedish (71 %), a lower education level (71 %), having never been married (75 %) and living in a rural municipality (78 %) [6, 17]. In most of these hard-to-reach groups, especially among women with a lower education level, the brush device seemed to reach slightly higher attendance rates by self-sampling, but the differences were non-significant (Table 2). In the different mother tongue groups, the difference in overall self-sampling participation rates was not as marked as in previous Finnish studies where as high as two-fold rates between different mother tongue groups could be seen, [5, 6, 17], and in the lavage group no difference between mother tongue groups was seen (Table 1). This is encouraging, and might be a reflection of self-sampling becoming more familiar to the women to be screened.

HrHPV positivity rates did not differ significantly between the devices, being 7.2 % with the lavage device and 5.9 % with the brush device, but the overall positivity rate was lower than the approximately 12 % observed in the previous Finnish self-sampling studies that also used HC2 [6, 17]. Regardless of careful inspections, no analysis-related reason for the lower hrHPV prevalence was found. Further no explanation was found by comparing the positivity rates of different municipalities, i.e., regional results. The observed hrHPV prevalence was in fact closer to the 8 % hrHPV prevalence by HC2 amongst women participating in the routine screening [25]. This may be a result of simple coincidence due to limited size of the study cohort or reflect to a low-risk population taking part by self-sampling this particular year. The sensitivity to detect CIN2+ lesions of HPV testing on self-taken samples is around 80 % and is somewhat lower compared to the clinician-taken samples when signal-based assays are used and thus further attention to the analytical validity aspects with self-sampling is required [26, 27].

The loss of follow-up, 36 % (8/22; 3/12 in the lavage group and 5/10 in the brush group), was higher than previously observed in Finnish studies [6, 17]. This was due to the non-compliance of women referred to a Pap smear (64 %) as the non-compliance rate to a colposcopy referral remained similar to previous experiences (9 %). However, the actual fall out rate may not be as high as reported: some women may have attended to their follow-up visits outside the organized screening program. In addition, three (14 %) of the hrHPV positive women moved to a municipality not participating in this study or abroad and thus their later health care records were therefore no longer available. Previously, highest compliance rates to follow-up Pap-smears in Finland, 79 %, were seen in a study that used pre-assigned appointment times in the invitations [6], making this the recommended invitational protocol if Pap-smear triage after self-sampling is used in the future.

Opportunistic Pap testing is extensive in Finland: 60 % of the Pap test taken for screening purposes are taken outside the organized program and the overall screening coverage in Finland is nearly 90 % when both organized and opportunistic tests are taken into account [28]. Opportunistic tests are not recorded in common databases in Finland, and were thus not available for those who did not respond to the questionnaire. Thus, even though the reminder letter and self-sampling increased the screening attendance, the exact effects on the overall screening coverage could not be calculated among all invited - this is a clear limitation of the study. The effect on the screening coverage remained smaller than the increase in the attendance, as only 20 % of the self-sampling participants were under-screened.

The response rate to the questionnaire was high in both groups, the demographic profile corresponded to the study cohort as a whole and the results thus give a reliable picture on previously non-attending women’s views on self-sampling. Self-sampling was regarded as easy with both devices by almost all participants who answered the question. Negative experiences (insecurity, discomfort, pain, embarrassment and fear) were reported rarely, but slightly more often with the brush device (Fig. 3). These minor differences in the self-sampling experiences between the sampler devices were however statistically non-significant and overall they did not seem to affect the willingness to participate with the brush device, as the self-sampling participation rate was higher in the brush device group. However, in the limited population of previously under-screened self-sampling participants (*n* = 67), self-sampling was more often regarded easy with the lavage device and some negative feelings were more often reported with the brush device (Table 3). This may reflect better acceptability of the lavage sampler in this high risk population, but the small number of observations clearly limits the wider generalization.

The previously observed higher prevalence of insecurity, fear and anxiety during sample taking amongst women with mother tongue other than Finnish or Swedish [15] was not observed in this study. None of the women in this language group reported having experienced fear or anxiety. Insecurity was reported by 33 % (2/6) of the lavage device participants but by none of the brush device participants. Amongst Finnish or Swedish speaking women insecurity was reported by 20 % (29/142) in the lavage group and 24 % (36/149) in the brush group. Mistrust on one’s ability to take the self-sample correctly and/or in the test result, often expressed as a barrier to self-sampling in previous studies [21–23, 29, 30], was not observed in this study. 93 % of women in both groups reported having felt confident about taking the sample correctly and 90 % of women in the lavage group and 85 % in the brush group stated that they trust the test result. Further, the mistrust in one’s ability to collect the sample, was rarely expressed as a reason to decline the self-sampling after receiving the sampler device, but slightly more often in the brush-group (17 %; 4/24 vs 8 %;2/25).

This study was conducted in a diverse set of Finnish municipalities where major invitational factors influencing the participation rates, personal invitations with pre-assigned appointment times and reminder letters to non-attendees, are already used. Previous self-sampling experiences in Finland were obtained with a lavage sampler that is no longer available in the market. Thus the current results of no significant differences between the sampler devices in overall attendance rate or user comfort allow for wider generalization of the previous results in further planning of invitation protocols. As an essential aspect of hrHPV-testing on self-taken samples is the heterogeneity between hrHPV-testing methods [26, 27], the choice of a self-sampling method for the Finnish program can thus be based on a clinically validated and cost-effective pair of a sampling device and a testing assay without the fear of losing women due to a less acceptable device.

## Abbreviations

ASC-US: Atypical squamous cells of unknown significance
CI: Confidence interval
CIN3: Cervical intraepithelial neoplasia, dysplasia gravis
HC2: Hybrid capture 2
hrHPV: High risk human papillomavirus
LSIL: Low grade squamous cell lesion
RR: Relative risk
